# What’s in a Color? The Unique Human Health Effects of Blue Light

**DOI:** 10.1289/ehp.118-a22

**Published:** 2010-01

**Authors:** David C. Holzman

**Affiliations:** **David C. Holzman** writes on science, medicine, energy, economics, and cars from Lexington and Wellfleet, Massachu-setts. His work has appeared in *Smithsonian*, *The Atlantic Monthly*, and the *Journal of the National Cancer Institute*

In 1958, J. Woodland Hastings and Beatrice M. Sweeney tested the ability of different wavelengths of light—corresponding to different colors—to shift the circadian rhythm in the photosynthetic marine dinoflagellate *Gonyaulax polyedra*. The greatest power to reset the organism’s daily meter lay in the blues, with a precipitous decline into the greens and a modest boost in the reds.

Hastings and Sweeney’s paper, published in the December 1958 *Biological Bulletin*, gathered dust for decades. No one thought these findings might hold any relevance for humans, whose circadian rhythms were then widely believed to be relatively insensitive to light.

But scientific discoveries in the past two decades have changed all that. Not only does light reset the human circadian rhythm, but the same blue light that has the strongest impact on dinoflagellates has equal power to reset our own clocks—although most visible wavelengths can reset the clock, the blues do the job with the greatest efficiency.

Now researchers are finding increasingly that an out-of-phase circadian rhythm is a health hazard. “Maintaining synchronized circadian rhythms is important to health and well-being,” says Dieter Kunz, director of the Sleep Research and Clinical Chronobiology Research Group at Charité–Universitätsmedizin Berlin. “A growing body of evidence suggests that a desynchronization of circadian rhythms may play a role in various tumoral diseases, diabetes, obesity, and depression.”

Shift workers, whom Kunz calls “a model for internal desynchronization,” are known to experience increased morbidity and mortality for a number of diseases, including cardiovascular disorders and cancer. In fact, in 2007, the World Health Organization decreed that shift work is a risk factor for breast cancer, and on that basis, in 2009, the Danish government began compensating some female shift workers with breast cancer.

At the same time, researchers have repeatedly shown that bright white light has the power to mitigate depression and other maladies of mood. An emergent recent literature suggests that blue light may be particularly potent for such applications.

## Unraveling the Mysteries of Circadian Rhythm

In the absence of normal cues of nightfall and daybreak, the circadian rhythm “runs free” and has been found to average about 24.25 hours. Night owls’ endogenous cycles typically run slightly longer, and morning people may cycle short of 24 hours. Exposure to the normal day/night cycle keeps people entrained (or aligned) on 24 hours through daily resetting, or switches them to new cycles when they cross time zones, almost the way you might reset your watch.

For many years it was thought that social interaction was the major force involved in resetting humans’ internal clock—in this regard, uniquely among the kingdoms of life, humans were thought to be relatively insensitive to light. In one study, researchers at the Max Planck Institute for Behavioral Physiology, led by Jurgen Aschoff and Rütger Wever, built a soundproof underground bunker in which subjects could be insulated from time cues. They even wrapped the bunker in copper wire, like the coils on an electric motor, to prevent the possibility that external electromagnetic forces, which might vary with time of day, might somehow cue time. Subjects’ responses to potential external cues or to their own endogenous rhythms were assessed by observing their sleep/wake cycles and various physiologic measures that cycle with circadian rhythm, such as body temperature, which rises during the day and falls at night.

Subjects were exposed to external circadian cues for 9 days, with light, temperature, and noise ebbing and flowing on 24-hour cycles. Under these circumstances, subjects showed 24-hour rhythms, as described in the January 1970 issue of the *European Journal of Physiology*. Then all external cues were removed. Subjects ate and turned lights on and off when they felt like it. Under these “free-running” conditions, the typical subject’s sleep/wake cycle exceeded 24 hours. Once the experimenters re-imposed the 24-hour cycle of light and dark, however, subjects’ bodies re-established a 24-hour circadian rhythm. During the 24-hour light/dark cycles, the researchers sounded a gong as they replaced light with darkness, to remind subjects of daily procedures such as collecting urine. But at some point, the gong malfunctioned; at the same time, one subject’s circadian rhythm ran free. When the experimenters discovered this, they conducted new experiments that appeared to show free-running rhythms only in the gong’s absence, says Czeisler. This finding convinced the researchers that social cues were critical to entraining the human circadian rhythm.

An incident 6 years later challenged that concept in Czeisler’s mind. In 1976, Czeisler’s advisor attended a closed meeting of some 18 top circadian scientists at the Max Planck Institute, and Czeisler, then a graduate student, tagged along. As the scientists toured an apartment in the bunker that was illuminated by table lamps, Czeisler asked a question that he says in retrospect seems ridiculous but that helped turn the field on its head. “What’s it like when it’s dark in here?” he asked. “‘It *is* dark in here,’ Dr. Wever responded,” Czeisler says.

It turned out that by “dark in here” Wever meant the overhead fluorescents—which represented daylight when the researchers turned them on each morning—were turned off. The table, kitchen, and bathroom lamps, which subjects could control themselves, weren’t believed to influence circadian rhythms. But apparently they did.

From 1980 to 1987, a series of papers came out that changed the field’s thinking on how human circadian rhythms are entrained, says George C. Brainard, director of the Light Research Program at Jefferson Medical College of Thomas Jefferson University. One set of papers, the first of which was published by Alfred Lewy, then a staff psychiatrist at the National Institute of Mental Health, demonstrated convincingly that bright white light at an intensity of 2,500 lux could have an acute effect on suppression of melatonin secretion, which is a marker of circadian sensitivity. Lewy’s work led to the use of bright light to treat mood disorders.

Czeisler’s first paper on circadian entrainment appeared in the August 1981 issue of *Photochemistry and Photobiology*. He demonstrated that light/dark cycles, not social interaction, entrained the circadian rhythms in 2 male subjects living in a specially constructed apartment devoid of potential time cues. In one phase of the experiment, the subjects were told the light/dark cycles would be more or less random. In reality, the subjects selected their own cycles, as the researchers turned the lights off when the subjects went to bed and on when they awoke. Under these circumstances, the subjects’ circadian rhythms ran free.

Subsequently, the researchers imposed 24-hour light/dark cycles, alternately advancing and retarding the clock—giving the subjects a light regime that was later or earlier, respectively, than the actual time—and then holding it constant. In each case, entrainment ensued.

In a paper published 8 August 1986 in *Science*, which Brainard calls a “landmark,” Czeisler showed that exposure to carefully timed bright light over several days could reset the circadian rhythm very precisely, the way one might reset a watch, even when the subjects’ bedtime was held constant.

Then, in the 1990s, some studies of circadian rhythm in blind people began showing results that begged big questions about unknown pathways for light perception. Some blind individuals, particularly those whose eyes had been removed, showed abnormal, free-running circadian rhythms with attendant sleep disorders, as one would expect for the sightless. Others who still had their eyes showed normal circadian rhythms. Czeisler was able to suppress melatonin secretion and shift circadian rhythm in the latter patients by exposing them to bright light. “That just blew us away,” he says.

But it didn’t blow away skeptical journal editors. One who rejected the paper said, “These people aren’t really blind, they are lying,” according to Czeisler, despite the subjects’ failure to perceive a neuro-ophthalmologist’s brightest light when he shined it directly into their eyes. After 5 years and 20 rejections, the *New England Journal of Medicine* published the paper 5 January 1995 after first making Czeisler test more subjects and cover both their eyes and their whole bodies, “just in case light might be penetrating some other body part.”

## The Story of a New Receptor

A major milestone came with the 1998 discovery of melanopsin retinal ganglion cells, a new type of photoreceptor in the eye. These cells provide signals to the suprachiasmatic nucleus (SCN), the brain’s master clock. They project to many other brain regions as well, influencing myriad aspects of human physiology. Moreover, research would show, they are uniquely sensitive to blue light.

The discovery of a new photoreceptor was largely unanticipated given that the eye’s anatomy had been well described for more than a century. But Russell Foster, now head of the Nuffield Department of Ophthalmology at the University of Oxford, United Kingdom, wasn’t all that surprised. In the early 1980s, Foster was researching avian circadian tracking. It had long been known that birds use neither eyes nor pineal gland for circadian entrainment, but had other, anomalous photoreceptors deep in their brains. By the early 1990s he had begun work on fish and mammalian circadian tracking.

In his early mammalian experiments, Foster tested the ability of light to disrupt circadian rhythms in blind mice with rods and cones that were badly damaged from a genetic disorder. The mice entrained normally, so he hypothesized that mammals, like birds, might also have nonvisual photoreceptors. To test that hypothesis, he engineered mice with rodless, coneless eyes. “To our intense pleasure, regulation of the body clock seemed perfectly preserved,” he says. “We were definitely dealing with a new receptor.”

As a graduate student in Foster’s laboratory at the University of Virginia in the early 1990s, Ignacio Provencio, now an associate professor in the university’s Department of Biology, had tried frequently, creatively, and ever vainly to identify the photoreceptors that reset the SCN. Later, as a postdoctoral researcher at the Uniformed Services University in Bethesda, Maryland, in what he viewed as a detour from these efforts, he studied dermal melanophores, photosensitive skin cells from the frog *Xenopus laevis*. The melanophores turn dark when illuminated and light in darkness; they probably function as camouflage, says Foster. The researchers suspected that opsin-triggered photopigments mediated the color change, and Provencio cloned a new photopigment dubbed “melanopsin” from the frog skin cells.

Provencio searched histologic sections of frog tissues for melanopsin, ultimately identifying the compound in eye and brain tissue in research he published in the 6 January 1998 issue of *Proceedings of the National Academy of Sciences*. He then identified homologs in the eyes of mice and humans, and published the findings in the 15 January 2000 *Journal of Neuroscience*. Sensing paydirt, Provencio found the mammalian homolog was housed in a rare subtype of retinal ganglion cells, while David M. Berson, a professor of ophthalmology and visual sciences at Brown University, first determined the location of the melanopsin retinal ganglion cells within the eye. These cells, which are found in front of the retina, process signals from rods and cones, sending them on to the brain’s visual centers. But the question remained: did melanopsin provide this light sensitivity?

In the December 2001 issue of *Nature Neuroscience*, Josh Gooley, then a first-year Harvard graduate student, showed in rats that melanopsin cells connect to the SCN. Berson, in an effort to prove these cells really are sensitive to light, disconnected the rods and cones from the melanopsin cells so the light sensitivity of the former would not affect his results. Then he plugged electrodes into the melanopsin cells. Under bright light, the electrodes signaled the melanopsin cells’ reaction, as reported in the 8 February 2002 issue of *Science*.

In work published 3 July 2003 in *Nature*, King-Wai Yau and Samer Hattar of The Johns Hopkins University knocked out the melanopsin gene in a rodless, coneless mouse. The mice could not be entrained, “providing the last crucial bit of evidence linking melanopsin to circadian behavior,” says Foster.

Meanwhile, other researchers explored the neural pathways that arise from the melanopsin cells. Roughly 40% of these cells’ axons project to the SCN. Hattar, an assistant professor in the Solomon H. Snyder Department of Neuroscience, traced others to regions of the brain that are involved in, among other things, speed of circadian re-entrainment, light’s effects on activity levels, sleep regulation, and hormone regulation. Such connections project to the brainstem, to the limbic system (including the amygdala, from whence fear springs), and to the cerebral cortex (the mainspring of such cognitive functions as language, analytic thought, and long-term memory).

## Blue Light Special

A parallel line of research, meanwhile, delved into the qualities unique to blue light. From 1995 until 2001, Brainard and his colleagues tested 72 healthy men and women in more than 700 experiments to determine the strongest wavelength for suppressing melatonin secretion. The result confirmed a slightly earlier Japanese study on mutant mice, showing that the blues are the most important wavelengths for entraining the circadian system. Cones, the color receptors, have a peak sensitivity in the greens, at 555 nm. For the rods, the peak comes at 507 nm. Across 10 published studies on humans, rodents, and monkeys, the peak sensitivity of the melanopsin receptors appears to span 459–485 nm, says Brainard.

Researchers have shown in humans that light influences hormone secretion, heart rate, alertness, sleep propensity, body temperature, and gene expression. Moreover, in such studies, blue wavelengths have been found to exert more powerful effects than green wavelengths. In experiments published in the September 2003 issue of *The Journal of Clinical Endocrinology and Metabolism*, Brainard, Czeisler, and Steven Lockley, an assistant professor of medicine at Harvard Medical School, compared suppression of melatonin in humans during 6.5 hours of nighttime exposure by monochromatic light at 460 nm, the peak sensitivity of melanopsin cells, with 555 nm, the peak sensitivity of the visual system. The blue wavelength suppressed melatonin for about twice as long as the green.

In other experiments, blue also proved more powerful in elevating body temperature and heart rate and in reducing sleepiness, according to Gilles Vandewalle, of the Center for the Study of Sleep and of Biological Rhythms at the University of Montréal. “[P]erformance improves acutely after the onset of light exposure, both at night and during the day,” Vandewalle and colleagues wrote in a review in the October 2009 issue of *Trends in Cognitive Neuroscience*. Electroencephalography has shown that light exposure reduces alpha, theta, and low-frequency activity, which are correlates of sleepiness. And Vandewalle showed that blue light proved superior to other wavelengths in enhancing responses in the left frontal and parietal cortices during a working memory task.

Experimental subjects had quicker auditory reaction times and fewer lapses of attention under blue light than green, says Lockley. In further experiments using electroencephalography, blue wavelengths suppressed sleep-associated delta brainwaves and boosted the alpha wavelengths, which are related to alertness. “This means you might be able to use short [blue] wavelengths as a sleepiness countermeasure,” Lockley says.

Hattar says no previous experiment has determined whether the ill effects of shift work are due to light stimulation at the wrong time of day, to the circadian clock’s being out of phase, or to a combination of the two. However, he adds, even as activating melanopsin photopigment during the day is believed to be beneficial, it could be bad to activate it at night.

The investigators who study bright light to tame mood disorders would agree. Lewy, now director of the Sleep and Mood Disorders Laboratory at Oregon Health & Science University, succeeded in suppressing melatonin in humans by applying bright light of 2,500 lux. This is much brighter than indoor lighting, but much less so than a cloudy day. Similar experiments had succeeded in animals, but had failed in humans. That was probably because in both cases only tepid illumination was used, Lewy reasoned; the success in animals probably resulted from laboratory animals’ lack of exposure to the bright outdoors.

That suggested to Lewy that “humans might have seasonal rhythms cued to natural photoperiod,” which he says would be insensitive to indoor lighting. And that led to treating seasonal affective disorder with bright light. The literature on the efficacy of blue light for treating this disorder is just now beginning to develop, and Brainard says much more work needed to confirm whether blue light is more potent than broad-spectrum white light.

Investigations of treatment of other mood-related maladies with bright light followed. Daniel Kripke, an emeritus professor of psychiatry at the University of California, San Diego, thinks bright light, particularly blue wavelengths, may also prove useful for treating premenstrual depression and bulimia, and he says there is preliminary evidence it might be useful for anxiety. And researchers at Case Western Reserve University, led by Patricia Higgins, an associate professor of nursing, are testing bright blue lights in a long-term care facility for patients with dementia. Very preliminary results “show promise in raising activity levels during daytime hours and increasing sleep at nighttime,” she says.

But blue’s benefit, and its detriment, are both a matter of timing. In one experiment, Kunz showed that exposing healthy subjects to 30 minutes of 500 lux polychromatic blue light an hour before bedtime, in their natural home environment, delayed the onset of rapid eye movement sleep by 30 minutes. The implications of that finding have yet to be determined, says Kunz. But the melanopsin receptors are particularly sensitive during the evening and nighttime hours, so “I’m pretty sure that at least many of the sleep disorders we are facing epidemically are related to evening or nighttime light,” he says. According to the National Center on Sleep Disorders Research, sleep-related problems affect 50–70 million U.S. men and women of all ages.

Kunz and others also suspect that outdoor artificial night lighting aggravates circadian disruption, although he says there is a dearth of human data on the subject. [For more information about artificial light at night, see “Missing the Dark: Health Effects of Light Pollution,” *EHP* 117:A20–A27 (2009).]

Kunz believes rapidly increasing knowledge concerning the circadian timing system and the coordination of physiologic and psychologic processes on the one hand as well as the increasing understanding of the mechanisms of circadian entrainment will induce a substantial change in our daily living. “The major aim will have to be to strengthen the circadian timing system which can be achieved by increasing the blue portion in artificial light during daytime and by reducing the same blue portion of artificial light during the night and evening hours,” he says. “Increasing the blue portion of artificial light may improve performance and learning ability in school kids and employees working indoors, and health will be improved in patients staying at nursing homes or hospitals.” On the other hand, he adds, a reduction of the blue portion in artificial light during nighttime hours could protect shift workers against disorders such as cancer and cardiovascular disorders as well as reduce sleep disturbances and their consequences among the general population.

## Coming Full Circle

The irony of blue as an environmental agent is that before the industrial age, it was merely a color. The unnatural lighting conditions we created turned it into both a potential hazard and a treatment for the ailments it brought about. In addition to the traditional architectural values of visual comfort, aesthetics, and energy efficiency, Brainard says architectural lighting must be redesigned to account for its biological and behavioral impact on humans. “Ultimately that should improve people’s health and well-being in the built environment,” he says.

“Some people consider the progress in the field of light and health over the last couple of years as the most important light induced innovation since the invention of the light bulb,” says Kunz. “Fascinating times are ahead of light industry, clinical chronobiologists, and architects, to mention just a few. By optimizing lighting regimes we will be able to improve health, save energy, and improve learning and performance.”

## Figures and Tables

**Figure:**
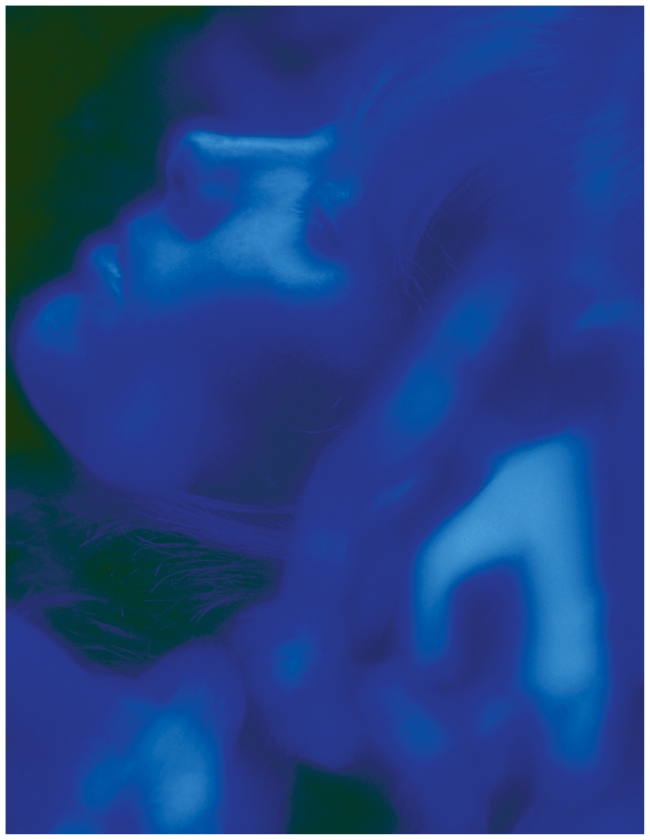
**The 1998 discovery** of a new photoreceptor in the eye—which later turned out to be especially sensitive to blue light—revolutionized the way we think about how circadian rhythm is entrained. Today we understand that blue light has many unique physiologic effects.

**Figure:**
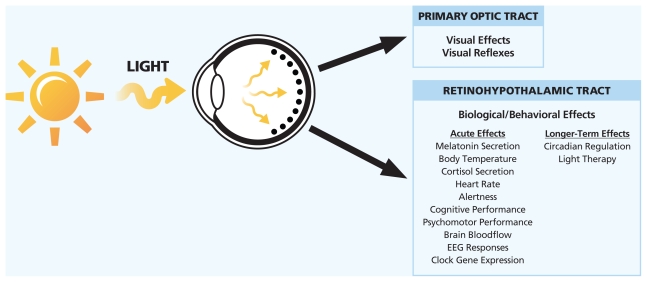
**Light: An Influential Environmental Agent** **Light** acts on the body by two pathways: the primary optic tract governs visual perception and responses whereas the retinohypothalamic tract governs circadian, endocrine, and neurobehavioral functions. The retinohypothalamic tract is most sensitive to blue light stimulation—energy in the wavelength of roughly 459–485 nm. Source: Benjamin Warfield and George Brainard/Thomas Jefferson University. Adapted by Matthew Ray/EHP.

**Figure:**
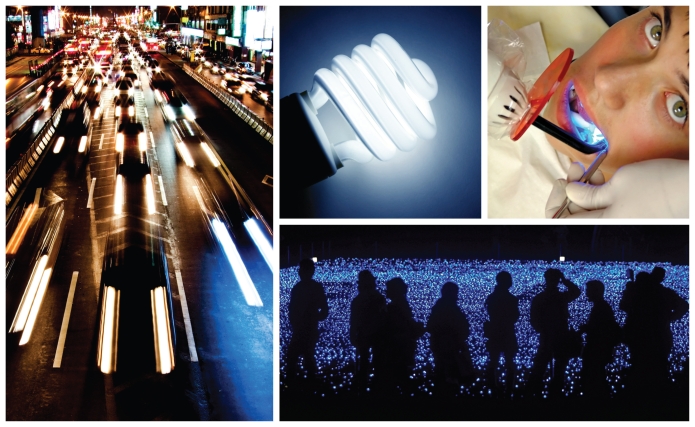
**Blue light**, subtle and dramatic, surrounds us, its special properties serving many purposes. When it comes to light perception, glare and brightness are both functions of wavelength; the short wavelength of blue light appears relatively bright to human eyes, making this among the most energy-efficient colors of light to produce. The bright bluish light emitted by high-intensity discharge headlamps thus increases visibility while using less energy than halogen headlamps, but that brightness also can heighten glare for oncoming drivers, particularly elderly drivers, who may already have trouble seeing at night. Now-ubiquitous compact fluorescent lamps (CFLs) similarly produce more light with less energy compared with incandescent lamps, and the bluer the CFL (“daylight” bulbs have the bluest color balance), the more energy efficient. More dramatic blue light is found in dental offices, where blue curing lights are used to harden amalgam material (orange goggles and filters provide eye protection against the intense light). The specific wavelength and intensity of the curing light stimulates a photoinitiator in the amalgam to decompose and initiate polymerization of the compound. But don’t think blue light is all work and no play—some, like that cast by the sea of Christmas lights at Tokyo Midtown, serves little purpose other than sheer enjoyment.

**Figure:**
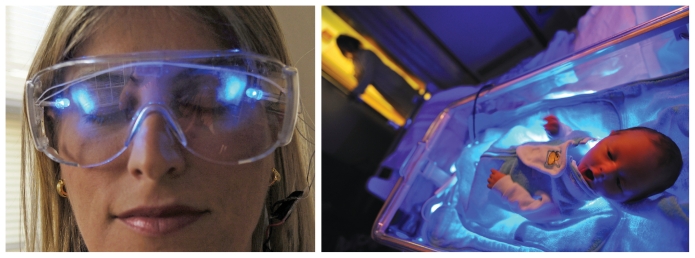
**Blue light**–emitting goggles, panels, and other devices are used to treat problems such as sleep disorders, jet lag, seasonal affective disorder, and premenstrual syndrome. But blue light doesn’t work solely through ocular stimulation; the shorter wavelengths can penetrate skin—this is how blue light is used to treat neonatal jaundice, in which the infant’s liver is unable to clear the normal hemolysis by-product bilirubin. Bilirubin builds up in the blood and enters body tissues, making the eyes and skin appear yellow. Blue light penetrates the skin and converts bilirubin into forms that can dissolve into the blood and be excreted in urine. The process repeats as untreated bilirubin continues to deposit into tissues from the blood, until most or all the bilirubin is converted.

**Figure:**
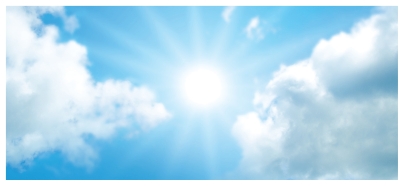

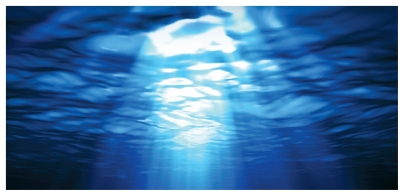
**Why blue?** Blue’s power to reset circadian rhythms is not intrinsic to the color. A photoreceptor for any color could have evolved to signal daylight to the suprachiasmatic nucleus. But the blues more easily penetrate the surface of the oceans—where life (and photoreceptors) likely first evolved—than do other visible wavelengths. The color balance of the sky may have helped to preserve blue’s clock-setting role throughout evolutionary history.

